# Cardiopulmonary resuscitation outcomes in a cancer center emergency department

**DOI:** 10.1186/s40064-015-0884-z

**Published:** 2015-03-01

**Authors:** Adam H Miller, Marcelo Sandoval, Monica Wattana, Valda D Page, Knox H Todd

**Affiliations:** Department of Emergency Medicine, University of Texas MD Anderson Cancer Center, 1515 Holcombe Blvd Unit 1468, Houston, Texas 77030 USA

**Keywords:** Cardiopulmonary arrest, End-of-life, Cardiopulmonary resuscitation, CPR, Survival to discharge, Return of spontaneous circulation

## Abstract

**Background:**

Cardiopulmonary resuscitation (CPR) after cardiac arrest is utilized indiscriminately among unselected populations. Cancer patients have particularly low rates of return of spontaneous circulation (ROSC) and survival to hospital discharge after CPR. Our study determines rates of ROSC and survival to hospital discharge among cancer patients undergoing CPR in our cancer center. We examined whether these rates have changed over the past decade.

**Methods:**

This IRB-approved retrospective observational study was conducted in our cancer center. The ED and cancer center provide medical care for ≥ 115,000 patients annually. Cases of CPR presenting to the cancer center for years 2003–2012 were identified using Institutional CPR and Administrative Data for Resuscitation and Billing databases. Age, gender, ethnicity, ROSC and Discharge Alive using a modified Utsein template was used to compare proportions achieving ROSC and survival to hospital discharge for two time periods: 2003–2007 (Group 1) and 2008–2012 (Group 2), using traditional Pearson chi-square statistics.

**Results:**

One hundred twenty-six cancer center patients received CPR from 2003–2012. Group 1 (N = 64) and Group 2 (N = 62) were similar; age (60 vs. 60 years), gender (63% vs. 58% male), and race/ethnicity (67% vs. 56% White). Proportions achieving ROSC were similar in the two time periods (36% Group 1 vs. 45% Group 2, OR = 1.47, 95% CI 0.72 - 3.00) as was survival to hospital discharge (11% Group 1 vs. 10% Group 2, OR 0.87, 95% CI 0.28 - 2.76).

**Conclusions:**

ROSC after CPR in cancer patients and survival to hospital discharge did not change over time.

## Background

Cardiopulmonary resuscitation (CPR) can be a life-saving intervention after cardiac arrest; however, the indiscriminate use of CPR among unselected populations and particularly among those with cancer confers only a small proportion of beneficial outcomes to hospital discharge (Varon and Marik 2007; Schneider et al. 1993; Bedell et al. 1983; Roberts et al. 1990; Berger and Kelley 1994; Stiell et al. 1992; Brown et al. 1992; Becker et al. 1991; Wenzel et al. 2004; Taran et al. 2012; Leak et al. 2013; Fu et al. 2011, 2012; Tan and Jatoi 2011; Ho et al. 2011; Myrianthefs et al. 2010; Hwang et al. 2010; Reisfield et al. 2006; Wallace et al. 2002). Upon the terminal event of dying in the United States, CPR is provided to all people without their implicit consent whether the event occurs in- or out-of the hospital. Only when patients give caregivers explicit instructions to withhold CPR is it not performed (Hansen-Flaschen 1991; Gleeson and Wise 1990; Varon and Marik 2007). In 1960, Kouwenhoven et al. (Kouwenhoven et al. 1960; Bernard et al. 2002) described closed chest message intending it to be administered primarily to otherwise “healthy patients” with reversible conditions who experienced sudden and unexpected cardiorespiratory arrest. Today, despite the universal application of CPR to all dying patients unless otherwise specified, in most cases and particularly in cancer patients, CPR temporarily prolongs the dying process by restoring spontaneous circulation (Schneider et al. 1993; Bedell et al. 1983; Roberts et al. 1990; Berger and Kelley 1994; Stiell et al. 1992; Brown et al. 1992; Becker et al. 1991; Wenzel et al. 2004; Wallace et al. 2002). During the last ten years, researchers have determined that cancer patients have a particularly low rate of return of spontaneous circulation (ROSC) and survival to hospital discharge after CPR (Varon and Marik 2007; Schneider et al. 1993; Bedell et al. 1983; Roberts et al. 1990; Berger and Kelley 1994; Stiell et al. 1992; Brown et al. 1992; Becker et al. 1991; Wenzel et al. 2004; Taran et al. 2012; Leak et al. 2013; Fu et al. 2011, 2012; Tan and Jatoi 2011; Ho et al. 2011; Myrianthefs et al. 2010; Hwang et al. 2010; Reisfield et al. 2006; Wallace et al. 2002). The survival to discharge rates for out-of-hospital CPR and in-hospital CPR in unselected CPR populations is 1% to 10% and 15% respectively (Schneider et al. 1993; Bedell et al. 1983; Roberts et al. 1990; Berger and Kelley 1994; Stiell et al. 1992; Brown et al. 1992; Becker et al. 1991; Wenzel et al. 2004) and for cancer populations it is <6% (Myrianthefs et al. 2010; Hwang et al. 2010). An increased emphasis on palliative care for cancer patients and the incorporation of patient goals of care in planning therapeutic interventions holds the promise that CPR might be used more selectively among those with cancer, thereby resulting in higher rates of ROSC and longer term survival after cardiac arrest. Our study objective was to determine rates of ROSC and survival to hospital discharge among cancer patients undergoing CPR in the emergency department (ED) and comprehensive cancer center. Additionally, we examined whether these rates have changed over the past decade.

## Materials and methods

The institutional review board of the University of Texas M.D. Anderson Cancer Center in Houston, Texas approved this retrospective observational study. This research was carried out according to institutional ethical guidelines that explicitly gave us permission to access these data retrospectively. The institution is a comprehensive cancer center that provides medical care for > 115,000 patients per year and has an annual ED volume of 22,000 patient visits annually. We identified cancer patients who underwent CPR (defined as patients receiving chest compressions) presenting to the ED and cancer center for years from 2002 to 2012. We identified cases using our institutional CPR database as well as a review of administrative data for resuscitation/CPR billing using CPT code 92950. We included patients who had cancer and received CPR initiated in the ED or who had CPR initiated outside the hospital and were then transported to the ED where it was continued. Patients were excluded if they did not have cancer (<5% of ED volume) or did not receive CPR. We abstracted data utilizing a modified Utstein template (Cummins et al. 1991) and recorded age, gender, ethnicity, evidence of ROSC and whether patients were discharged alive from the hospital. Each author performed a review of identified charts and completed a data tool. We compared proportions achieving ROSC and survival to hospital discharge for two time periods: 2002–2007 (Group 1) and 2008–2012 (Group 2), using traditional Pearson chi-square statistics.

## Results

**Figure 1:**
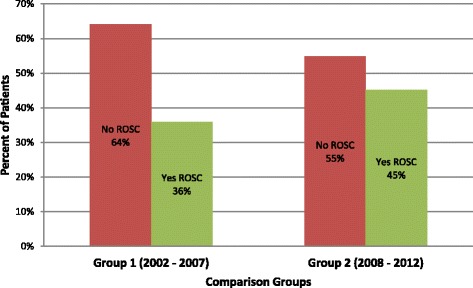
**Return of spontaneous circulation (ROSC) among 126 cancer patients undergoing cardiopulmonary resuscitation (CPR) in a comprehensive cancer center comparing Group 1 (2002 – 7) and Group 2 (2008 – 2012).**

**Figure 2:**
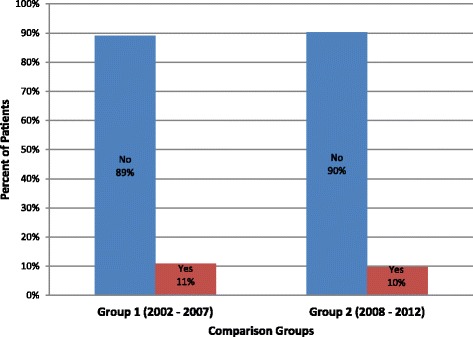
**Survival to discharge among 126 cancer patients undergoing cardiopulmonary resuscitation (CPR) in a comprehensive cancer center comparing Group 1 (2002 – 2007) and Group 2 (2008 – 2012).**

We identified 126 ED and cancer center patients who received CPR for cardiac arrest in our ED or cancer center from 2002–2012. Group 1 (N = 64) and Group 2 (N = 62) were similar with regard to age (60 vs. 60 years), gender (63% vs. 58% male), and race/ethnicity (67% vs. 56% White) (Table 1). Proportions achieving ROSC were similar in the two time periods (36% in Group 1 vs. 45% in Group 2, OR = 1.47, p = 0.29; 95% CI 0.72 to 3.00) (Table 2 and Figure 1). Similarly, survival to hospital discharge did not appear to change over time (11% in Group 1 vs. 10% in Group 2, OR 0.87, p = 0.82; 95% CI 0.28 to 2.76) (Table 2 and Figure 2).

**Table 1:** **Distribution of demographic characteristics of cancer patients undergoing cpr in the emergency department of a comprehensive cancer center**

**Demographic characteristic**	**Category**	**Total**		**Group 1 (2002–2007)**		**Group 2 (2008–2012)**	
		**No**	**%**	**No**	**%**	**No**	**%**
Gender	Male	76	60	40	63	36	58
	Female	50	40	24	37	26	42
	Total	126	100	64	100	62	100
Race	Black	34	27	16	25	18	29
	Hispanic	6	5	2	3	4	7
	Other	8	6	3	5	5	8
	White	78	62	43	67	35	56
	Total	126	100	64	100	62	100
Age (Years)	Mean	60		60		60	
	Median	62		63		61	
	Range	12-89		21-77		12-89	
Cancer Diagnos	Lung	21	17	12	19	9	15
	Leukemia	17	13	9	14	8	13
	Breast	15	12	9	14	6	10
	Lymphoma	10	8	5	8	5	8
	MultipleMyeloma	7	6	3	5	4	6
	Sarcoma	6	5	0	0	6	10

**Table 2:** **Return of spontaneous circulation (ROSC) and survival to hospital discharge among cancer patients undergoing cpr in the emergency department of a comprehensive cancer center**

**Event**	**Outcome**	**Total**		**Group 1 (2002–2007)**		**Group 2 (2008–2012)**		**P-value**	**Odds ratio (95% CI)**
		**No**	**%**	**No**	**%**	**No**	**%**		
ROSC	No	75	60	41	64	34	55	0.29	1.47(0.72-3.00)
	Yes	51	40	23	36	28	45		
	Total	126	100	64	100	62	100		
Survival to Discharge	No	113	90	57	89	56	90	0.82	0.87(0.28-2.76)
	Yes	13	10	7	11	6	10		
	Total	126	100	64	100	62	100

## Discussion

We found that the rates of ROSC after CPR in cancer patients trended toward improvement by a small amount over the past decade at our institution, though these changes were statistically insignificant. Any trend toward improved ROSC outcomes that may exist could result from improvements in CPR quality. However, the outcome of survival to hospital discharge has not changed over the two time periods studied. Dissimilar to the cancer CPR population, new technologies including the use of automated external defibrillators (AEDs), well delineated locations of AEDs for improved access, new resuscitation algorithms including cardiac compression rates, ventilation rates and volumes, performance of basic CPR before defibrillation, and therapeutic hypothermia have all been manipulated to maximize resuscitation efforts, demonstrating improvements in neurological recovery and outcomes in the unselected CPR population (Holzer et al. 2002; Yannopoulos et al. 2006; Wik et al. 2003; Hallstrom et al. 2004; Eliott and Olver 2007; Reisfield et al. 2006; Hwang et al. 2004). The lack of improvement in survival to hospital discharge in our study may suggest that CPR in cancer patients continues to be performed on an unselected cancer population, rather than being targeted toward subsets of cancer patients who are more likely to receive benefit. Reisfield et al. (2006) reported in 2006 that in 1,707 cancer CPR patients with solid tumor, the rate for being discharged alive to another facility was 7.1% compared to only 2% of those with a hematologic malignancy. Consistent with these data, Hwang et al. (2010) reported in 2010 that in 41 patients after out-of-hospital cancer CPR, the discharge alive to another facility in those with solid tumor was 18.0% compared to 12.5% in the hematologic malignancies. Ultimately however, the overall survival to discharge home for cancer CPR patients was 4.9%. Perhaps continued efforts to identify the specific type(s) of cancer that would benefit the most in terms of survival to hospital discharge must be further defined, thus, allowing end-of-life and advance care planning techniques to be more effective. Again dissimilar to the cancer CPR population, end-of-life and advanced care planning may have already contributed to improved outcomes of CPR in in the unselected CPR population by allowing those terminally ill in this general population group to choose an a priori do-not-resuscitate (DNR); effectively decreasing the effect size of those that would have worse outcomes (Sculier 1995). Therefore, selecting out the least likely to survive before CPR is required and excluding them from ever entering the sample calculation ultimately improves the total outcome.

Another area to exploit in the quest for improved CPR in unselected and selected CPR patient outcomes can be by improving communication and/or documentation (living wills and medical powers of attorney) and family education (the meaning of a DNR order). These interventions will allow the patient and loved ones of the patient to avoid transporting the patient to the hospital and not be given CPR when dying occurs (Varon and Marik 2007).

These study results reflect the other two studies referenced pertaining to cancer CPR outcomes whether in- or out-of-the-hospital. It is an important topic as cancer increases in frequency in the United States, and the discussion about end of life issues becomes more relevant, particularly the economic impact taking into account. The need to identify the cancer patient who will benefit the most from CPR and end-of-life interventions including DNR is of paramount importance moving forward.

### Limitations

A retrospective study of this type has numerous limitations including selection bias. Some potential cases may have been omitted if; the documentation did not include the CPT code we queried, CPR was not documented on the medical record; or the CPR designation was missing from the institutional logs and databases. Some of the Utstein type data were unavailable for pre-hospital cases in the databases that we queried including number of resuscitation attempts, bystander witnessed or unwitnessed arrest, bystander CPR or defibrillation or cardiac vs. non-cardiac etiology of arrest. As such we could not comment on these factors and their effect on survival. Furthermore, the small sample size of this single center study compared to the other study we referenced (Reisfield et al. 2006) makes it difficult to generalize it to other cancer populations, nor does it enable sufficient power to conclude a valid statistical outcome.

## Conclusions

In our study population, change in ROSC between the two groups was statistically insignificant though trended toward improvement. Survival to hospital discharge after CPR between the two groups was not changed when proportions were compared between 2002–2007 and 2008–2012 groups. Larger study populations, for whom we have more information on potential confounding factors, will be required to more definitively answer our study question.
